# Experimental Behavior of Thin-Tile Masonry under Uniaxial Compression. Multi-Leaf Case Study

**DOI:** 10.3390/ma14112785

**Published:** 2021-05-24

**Authors:** Joan Llorens, Miquel Àngel Chamorro, Joan Fontàs, Manuel Alcalà, Marc Delgado-Aguilar, Fernando Julián, Miquel Llorens

**Affiliations:** 1 CATS Research Group, Department of Architecture and Construction Engineering, University of Girona, Avda Maria Aurelia Capmany 61, 17071 Girona, Spain; mangel.chamorro@udg.edu; 2 UdiGitalEdu Research Group, Department of Architecture and Construction Engineering, University of Girona, Avda Maria Aurelia Capmany 61, 17071 Girona, Spain; Joan.fontas@udg.edu; 3 LEPAMAP-PRODIS Research Group, Department of Organization, Business Management and Product Design, University of Girona, Avda Maria Aurelia Capmany 61, 17071 Girona, Spain; Manuel.alcala@udg.edu (M.A.); Fernando.julian@udg.edu (F.J.); 4 LEPAMAP-PRODIS Research Group, Department of Chemical and Agricultural Engineering and Agrifood Technology, University of Girona, C/Maria Aurèlia Capmany 61, 17003 Girona, Spain; m.delgado@udg.edu; 5 AMADE Research Group, Department of Mechanical Engineering and Industrial Construction, University of Girona, Avda Maria Aurelia Capmany 61, 17071 Girona, Spain; miquel.llorens@udg.edu

**Keywords:** brick, thin-tile, compressive strength, mechanical properties, experimental analysis, thin-tile vault

## Abstract

In this study, experimental analysis on the compressive strength of multi-leaf thin-tile masonry is presented. A compressive strength test was carried out on thin-tile, mortar and 48 specimens with two- and three-leaf thin-tile masonry. The results obtained were compared with literature on brick masonry loaded parallel to a bed joint. Based on the results of this study, the failure mode presented the first crack in the vertical interface; this crack grew until the leaf was detached. From this point until collapse, lateral buckling of the leaves was generally observed. Therefore, the detachment compressive strength value was considered relevant. Up to this point, both masonries exhibit similar stress–strain behavior. The experimental values of the detachment compressive strength were compared with the values calculated from the equation generally used in the literature to evaluate the compressive strength of brick masonry. From the results obtained, the following conclusion can be drawn: This equation is only suitable for tree-leaf thin-tile masonry but with more relevant influence on the compressive strength of the mortar. This study concluded that only three-leaf specimens behave similarly to brick masonry loaded parallel to a bed joint. Finally, whether the failure mode was due to shear or tensile stresses in the vertical thin-tile-mortar interface cannot be identified.

## 1. Introduction

**Figure 1:**
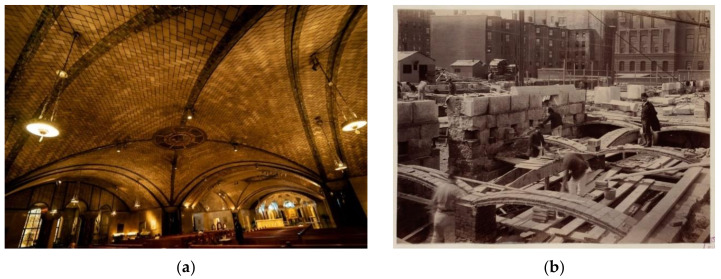
Examples of buildings with thin-tile vaults. (**a**) National Shrine of the Immaculate (**b**) Rafael Guastavino stands on a recently laid thin-tile arch along Boylston Street, construction of the McKim Building. (Boston Public Library Conception, Washington).

Structural elements, such as load-bearing walls, arch and vaults, built with ceramic masonry (Figure 1a), are common in historical buildings. In order to preserve such buildings, a careful study of their mechanical behavior is needed. A singular case is the masonry used to build the thin-tile vault. This is characterized by the use of several leaves of thin tile, placed flat between leaves of mortar approximately 10 mm thick (Figure 1b). In older vaults, the thin tile was bonded with gypsum or lime mortar, while cement mortar is commonly used in current structures. This structural element is characterized by being subjected to compressive stress, with the particularity of applying the load parallel to the face of the thin tile.

As stated by Gulli and Mocchi [1], to understand the structural behavior of the vault, there are mainly two approaches. The first is the equilibrium approach, which suggests that stability is achieved if the gravitational forces on the different pieces (stone or brick) of the vault are in equilibrium. The second is the elastic approach. This approach is based on the assumption that thin-tile masonry is a conglomerate material of monolithic nature as a result of mortar hydration. The most common approach for the equilibrium approach is the limit analysis based on the theory of plasticity proposed by Heyman [2]. The author defined the three fundamental assumptions, namely, (a) masonry is a compressively rigid material, (b) masonry has no tensile strength and (c) sliding between pieces of masonry cannot occur. Based on these assumptions, the upper and lower bound theorems of the theory of plasticity are useful to evaluate the load-bearing capacity of the vault. As stated in Rondeaux and Zastavni [3], the lower bond theorem could be defined as the load limit, calculated from a statically compatible distribution of internal forces and applied loads respecting yield conditions, is less than or equal to the collapse load. Conversely, the upper bond theorem could be defined as the load limit calculated from a kinematically compatible mechanism is greater than or equal to the collapse load. In this context, the predictable mode of failure of the vault is the formation of a sufficient number of hinges.

The elastic approach was first presented by Guastavino [4]. The author described the cohesive construction, similar to that built with a conglomerate material obtained by means of a transformation (hydration) from thin tiles and mortar. In this way, the new material could withstand tensile stresses. Different authors proposed models for elastic behavior. Thus, in Capozucca [5], a method of analysis based on the Sandwich behavior model proposed by Flügge [6] was developed. The author found that when the limit state of the mortar leaf was reached, the model collapsed due to shear forces. The author concluded that the Sandwich behavior model was suitable for the thin-tile vault. In Endo et al. [7], the authors analyzed the static and dynamic behavior of thin-tile vaults based on experimental tests and analytical models. In the compressive strength test, the detachment of the leaves on the specimens was observed. This detachment was achieved at values below the maximum compressive strength. In Benfratello et al. [8], specimens of thin-tile vaults obtained from portions of real structures through compressive and bending strength tests were analyzed. The authors compared two analytical material models (homogeneous and stratified) with experimental results. Based on the results obtained, researchers concluded that the stratified model better describes the behavior of the material and, therefore, of the whole structure.

The usual failure mode in thin-tile masonry is the collapse by the formation of a sufficient number of hinges [9,10,11]. Other failure modes are presented in the literature. Thus, in [7,8], on compressive strength test of a portion of the vault, the detachment of the leaves as failure mode was stated. Fiber-reinforced polymer FRP is commonly used for masonry reinforcement or repair [12]. In De Lorenzis et al. [13], the aim of this reinforcement was to prevent or reduce the formation of the hinges. The authors found that in these reinforced vaults, the failure mode was reached by local failures, such as crushing of the masonry, sliding of mortar joints or the detachment of the leaves. Other authors [14,15,16] presented studies on arches and vaults strengthened using composite materials, such as FRP reinforcement, among others, with a similar failure mode.

Although the failure mode of a thin-tile vault is normally by the formation of a sufficient number of hinges, other local failure modes can be obtained. Therefore, the compressive strength is a relevant issue when the failure mode of the vault is due to a local failure.

An often-studied type of masonry is the load-bearing brick masonry, commonly used to build walls. This masonry is characterized by the application of the load perpendicularly to the bed joints. In preliminary research, [17,18,19,20,21,22,23,24,25,26] analyzed the behavior of this masonry for several stress orientations with regard to the bed joints. In [27,28], it was concluded that, for normal and perpendicular stress, the stress–strain behavior was similar. The arrangement of the leaves in the thin-tile masonry could allow considering it as a particular case of masonry loaded parallel to the bed joints. In literature about brick masonry walls, it is common to establish compressive strength using the exponential Equation (1):

*f_k_* = *K*·*f_b_*^α^·*f_m_*^β^ (1)

where *ƒ_k_*, *ƒ_b_* and *ƒ_m_* are the compressive strength of the masonry, brick and mortar, respectively, while *K*, *α* and *β* are coefficients adjusted experimentally. The first corresponds with variations of the joint and brick used, while the other two act as corrective coefficients. Several authors and standards have defined these values for load-bearing walls [29,30,31,32,33].

The standards [34,35] indicate for masonry loaded parallel to the bed joints, the use of the same Equation (1). In this instance, the *ƒ_b_* value must be obtained in the same orientation as the load.

The goal of this study was to verify whether the proposed compressive strength exponential equation for masonry loaded parallel to the bed joints was applicable to thin-tile masonry. For this purpose, an experimental program on 48 specimens of two- and three-leaf thin-tile masonry built with two types of thin-tile and mortar were presented.

Based on the results obtained, the stress–strain behavior and failure mode of both masonries were compared. An analytical model based on equation exponential commonly used in brick masonry loaded parallel to bed joints was defined and compared with the experimental results. Finally, conclusions were drawn.

## 2. Materials and Methods

This study was part of an experimental test described in [36]. Within this campaign, two- and three-leaf specimens represented the thin-tile masonry. 

### 2.1. Thin-Tiles

**Figure 2:**
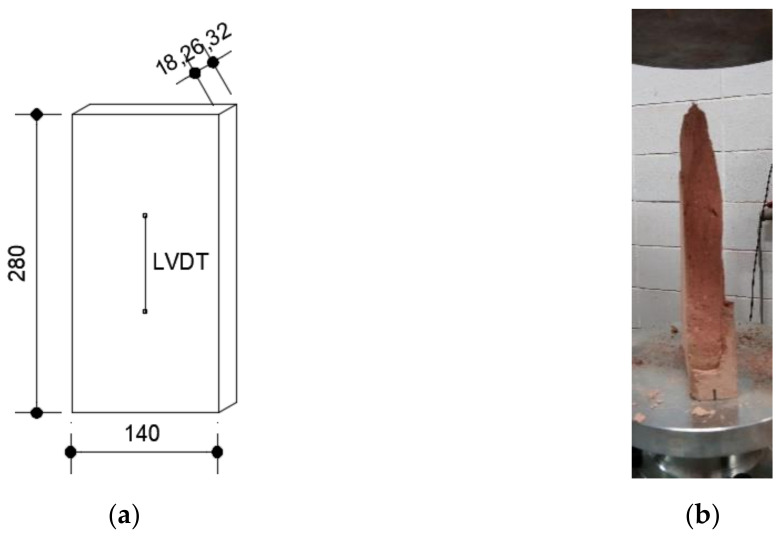
(**a**) The geometry of the specimen and the placement of LVDT; (**b**) the mode of failure (dimensions in mm).

Two types of thin tiles were used that are very common in the construction of thin-tile masonry. C1 thin tiles made using a traditional manufacturing process, i.e., manual manufacturing, air drying and burning in a wood-fired oven. C2 thin tiles made using an industrialized manufacturing process. The dimension of the thin tiles was 290 × 140 mm in order to guarantee the flatness of the faces of the specimens, and they were all cut to 280 mm in height. Two different thicknesses of the two- and three-leaf specimens were chosen. To identify the thin-tile specimens, 4 digits were used (CXYZ), where CX corresponded to the type of thin-tile (C1 or C2) and YZ to its thickness (18, 28 or 32 mm) (Table 1). Five specimens represented each thin-tile series. The thin-tile compressive strength (*f′_tt_*) was obtained, according to standard [37]. The load application was carried out with displacement control at a speed of 0.01 mm/s. Vertical displacement was recorded using a linear variable displacement transducer (LVDT) on each face of the thin tile. Figure 2a shows the geometry of the specimens and the placement of the LVDTs.

In order to determine the normalized compressive strength (*f_tt_*), the standards established two correction coefficients to be applied to the experimental values obtained. The first coefficient takes into account the slenderness of the specimen. The standard proposes the value of the coefficient for multiple slenderness and allows the linear extrapolation between adjacent values. In [36], the authors analyze the thinness of the specimen and conclude that failure occurs in the compressive failure range of the material. The second coefficient takes into account the conditioning of the specimen prior to the test. Air-drying was used in this study. Table 1 shows the average value and the coefficient of variation (CoV) of compressive strength (*f′_tt_*), normalized compressive strength (*f_tt_*) and Young’s modulus (*E_tt_*), calculated as a secant modulus at 1/3 of *f_tt_* in accordance with [38]. 

From the experimental results obtained, it can be drawn that the normalized compressive strength of C118 Thin-tile has a significantly lower value of C132. This can be associated with the manual manufacturing and burning process in a wood-fired oven without temperature control. Thus, the two types of thin tiles have different burning levels when placed together in the oven. On the other hand, the C1 thin tiles show clearly higher values than the C2 ones. As observed in Figure 2b, material failure is the precursor to collapse.

### 2.2. Mortar

In the construction of thin-tile masonry, a hydraulic lime mortar was traditionally used. Currently, the use of a Portland cement mortar is more common. Thus, two types of pre-packaged commercial mortars were used. The MP mortar is a Portland cement mortar with CEM II-42.5R Portland cement and marble sand with an expected 28-day compressive strength of 7.5 N/mm^2^. The MC mortar is a natural hydraulic lime mortar (NHL) with marble sand and silica of 3.5 N/mm^2^ at 28 days. The binder/sand ratio was 1:6, and the manufacturers indicated the water content (4–4.5 L and 4.5 L for MP and MC mortars, respectively).

The compressive strength of the mortar (*f_m_*) was obtained according to the standard [39]. Six 4 cm side cubic specimens represented each mortar series. The specimens were stored for 5 days in the molds within a polythene bag. The following 2 days in the same bag, but outside the molds, and until the test at a temperature of 20 ± 1 °C and relative humidity of 65 ± 5%. For a better representation of the mortar within two- and three-leaf masonry specimens, in each manufacture of these specimens, one series of mortar was made. Additionally, on the same day as the masonry specimens, cubic ones were tested.

Table 1 shows the average and the coefficient of variation in parenthesis of compressive strength and Young’s modulus of Portland cement mortar (MP) and natural hydraulic lime mortar (MC). Values of Young’s modulus of each type of mortar corresponds to values obtained in the previous study [36].

It can be observed a significant variation in the strength of each series of specimens was obtained, especially in the Portland cement mortar. Since it is a premixed mortar, the difference of compressive strength between the different series could be attributed to a variation of the water included to facilitate working with the mortar.

### 2.3. Compressive Strength of Thin-Tile Masonry

**Figure 3:**
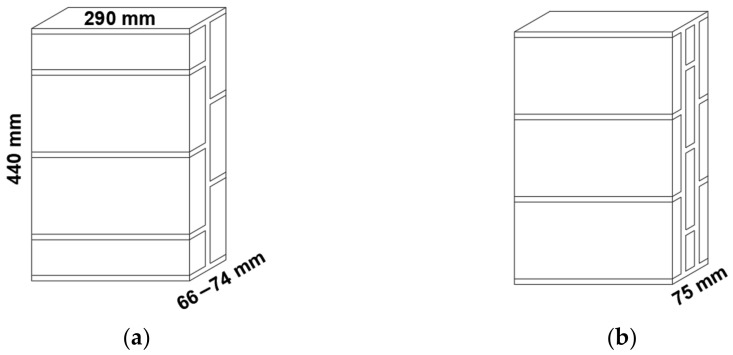
The geometry and arrangement of the thin tiles in the two-leaf (**a**) and three-leaf (**b**) specimens (dimensions in mm).

The masonry compressive strength test was conducted in accordance with the regulation [38]. The specimens were 440 × 290 mm^2^, and thickness were ranging from 66 to 75 mm depending on the thin-tile thickness and number of leaves (Figure 3). To ensure the verticality of the specimens, they were built in a horizontal position supported on the back face. To guarantee the flatness of the surface where the load was applied, the upper and lower face of the specimen was regularized with a 1 cm joint of the same mortar used for its manufacture. The specimens were identified with six digits (CVWXYZ), CV indicate the type of thin-tile (C1, C2), WX type of mortar (MP, MC), Y number of the leaves and Z position in the series. Thus, C2MP21 represents the first specimen with two leaves made using C2 thin-tile and Portland cement mortar. In order to reduce the variables to be analyzed, only one type of mortar has been used in each specimen.

Curing of the specimens involved covering them for the first 3 days with a polyethylene film. Subsequently, they were stored at a temperature of 15 °C and relative humidity of 65% until the test. In order to equalize the circulation of air on both sides of the specimen, halfway through the drying period, they were turned.

**Figure 4:**
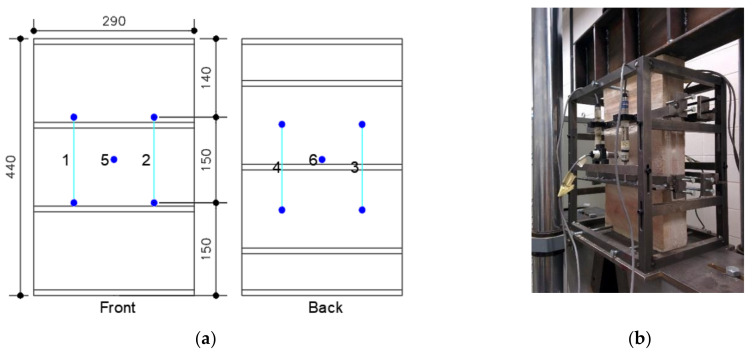
(**a**) The instrumentation diagram, placement of longitudinal and transversal LVDTs. (**b**) The test setup (dimensions in mm).

The compressive strength test was a similar process to the one used on the thin tiles but with displacement control. According to the regulation, the speed rate was defined at 0.002 mm/s to achieve collapse between 15 and 30 min. The displacement was recorded by two longitudinal linear variable displacement transducers (LVDTs number 1, 2, 3 and 4) and a transverse LVDT (5 and 6) on each side of the specimen (Figure 4). In accordance with the standard [38], the deformation of the specimen in the central third was recorded by the longitudinal LVDTs. On the other hand, the cross-section deformation of the specimen was recorded by transverse LVDTs. To allow the adaptation of the specimen to the setup, stress and strain values up to 0.5% of the peak compressive strength were discarded.

The hardening process time of hydraulic lime mortar is longer than that of Portland cement. In the literature [40,41], this process, and for lime mortars similar to those in this study, was established at around 90 days. For an adequate organization of the experimental tests, 84 days was established as the hardening process time for the specimens of this study.

## 3. Results

### 3.1. Failure Mode

**Figure 5:**
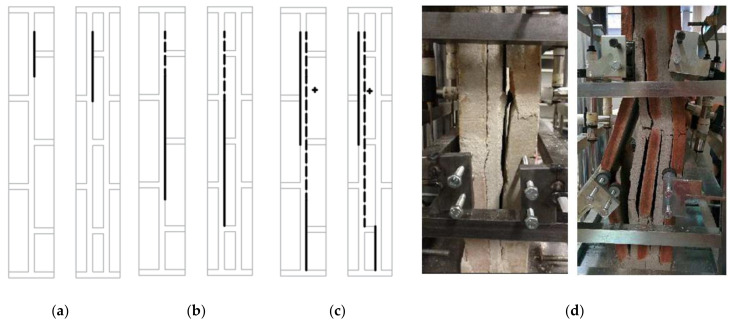
The failure pattern of the two- and three-leaf specimens. (**a**) The initial splitting, (**b**) crack crossing along the interface, (**c**) end of propagation and new crack appearance (detachment) and (**d**) bucking collapse of the specimen.

The development of the failure mode of the specimens in the compressive strength test is presented in (Figure 5). The new crack is represented by a continuous line, while the crack that appeared at previous stress levels is represented by a dashed line. The failure mode was initiated by splitting around the horizontal mortar joint of one of the thin-tile leaves, affecting one or two vertical thin-tile-mortar interfaces. As the stress increased, the crack was propagated through one of the interfaces until it reached the other end of the specimen, causing the leaf to detach. At which point, a new crack used to appear in another thin-tile-mortar interface. The increased stress led to the collapse of the specimen. In most cases, it is reached by buckling of the individual leaf, but in some two-leaf specimens, the collapse is achieved by crushing the thin tile. Several authors have reported a similar failure mode in masonry loaded parallel to bed joints [42,43,44,45].

### 3.2. Mechanical Properties

In this study, the stress at the moment of the detachment of one-leaf described in the failure mode was identified as detachment compressive strength. Table 2 shows the average values and coefficient of variation for peak (*f_kt_*), detachment (*f_ktd_*) compressive strength of thin-tile masonry and Young’s modulus (*E_kt_*) calculated as a secant modulus at 1/3 *f_kt_*. The horizontal LVDTs record the displacement in the thickness-axis. This displacement includes the deformation in the specimen due to the load but also the separation of the leaves. Thus, according to the values obtained, the coefficient of Poisson may not be computed accurately. On the other hand, in the horizontal width-axis, the specimen was built without any mortar joint; in this case, the values obtained would only represent the coefficient of Poisson of the thin tile.

According to the experimental values, the MC mortar specimens have a lower peak compressive strength than the MP mortar samples. For C1 thin tiles, this loss could be estimated as 31%, while for C2, the value ranged from 10% to 19%. On the other hand, the values for compressive strength due to detachment were 9% and 36%, respectively. 

In turn, the specimens built with C1 thin tiles presented higher values of peak compressive strength than those built with C2, being more notable in the specimens with two leaves.

From the comparison of the specimens built with similar mortar and thin-tile, it can be drawn that the detachment compressive strength of the two-leaf specimens has higher values than those of the three-leaf specimens. This difference could be estimated as an increase of 19% and 10% for specimens C1 and C2, respectively. According to these results, it seems that the stress was not similarly borne by the mortar and thin-tile leaves.

### 3.3. Stress-Strain Behaviour

**Figure 6:**
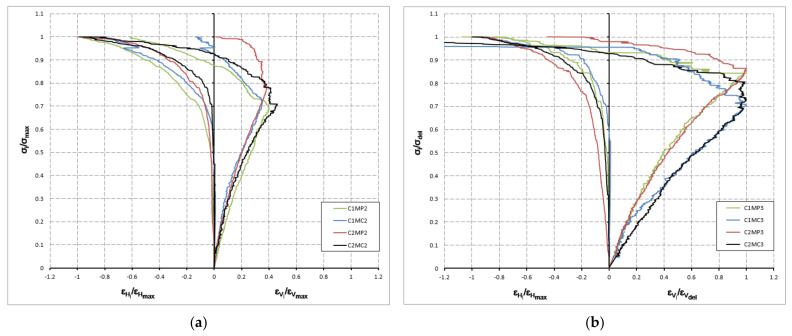
Normalized stress–strain behavior of two- (**a**) and three-leaf (**b**) masonry specimens.

Figure 6 shows the stress–strain diagram obtained from the compressive strength test of specimens with two- (**a**) and three-leaf (**b**). To improve the comparison of these diagrams, the normalized values are presented. On the vertical axis, the stress values were referenced to the peak compressive strength value of the specimen. On the horizontal axis, the vertical and horizontal strain values were referenced to the strain value for the peak compressive strength, with the exception of the vertical strain values of the three-leaf specimens, which were compared to the strain value for the detachment compressive strength (*ε_vd_*). To ensure their integrity, the LVDTs were removed once the maximum strain was exceeded. Therefore, the stress–strain diagram does not show the deformation in the descending branch.

The stress–strain diagrams in Figure 6 show a significant shift in slope at 70–85% of the maximum stress. This change coincides with the detachment of one of the leaves from the specimen. Therefore, in this study, the stress at this level has been referred to as the detachment compressive strength. Up to this point, the thin tile masonry presents the usual behavior of brick masonry. From this point on, the horizontal deformation remains the usual for brick masonry but presents an unusual elongation in the vertical deformation. In the failure mode, it was indicated that collapse is reached by buckling of the leaves. Thus, the LVDT’s attached to the exterior leaves a record, on the one hand, vertical shortening of the leaf due to tension but also an elongation due to buckling of the leaf.

Considering the influence of the constituent materials on the stress–strain diagram, it can be indicated that the horizontal deformation shows a clear influence of both materials. Thus, specimens constructed with MP mortar are softer than those constructed with MC mortar. As for the thin-tile, the C1 thin-tile shows higher deformation for the two-leaf specimens and C2 thin-tile for the three-leaf specimens. The vertical deformation of the three-leaf specimens indicates a remarkable influence regarding the mortar that is not observed for the two-leaf specimens.

## 4. Discussion

The Standards [34,35] propose the exponential Equation (1) to determine the compressive strength of brick masonry. According to these standards, when the load is applied parallel to the bed joints, the compressive strength of the brick (*ƒ_b_*) must be obtained from a sample where the brick is loaded in the same direction as the masonry.

Generally, in the literature, the values *K*, *α* and *β* are established from the experimental values of the compressive strength of piece, mortar and masonry. These values are computed by regression analysis based on the least-squares method of adjustment. To check whether Equation (1) is applicable to thin-tile masonry, Table 3 shows the values for and the coefficient of determination (R^2^) obtained from this study.

According to the results, and for two-leaf masonry, the *K*, *α*, *β* values found in this study showed a large variation with those reported in the literature [36,43]. On the other hand, the coefficient of determination indicated a very low level of confidence. Thus, it could be stated that Equation (1) is not adequate to determine the compressive strength of two-leaf thin-tile masonry. The values *K, α* and *β* for the three-leaf masonry would be in the usual range of the literature but with a greater influence of the mortar compressive strength. The stress–strain behavior also suggested such an influence. Consequently, Equation (1), and for values of this study, appears to be suitable to calculate the compressive strength for three-leaf thin-tile masonry.

The failure mode observed of thin-tile masonry was consistent with that described in the literature for masonry loaded parallel to the bed joints. In [43], the authors found that, for masonry loaded parallel to bed joints, if the difference in stiffness between mortar leaf and brick leaf is significant, premature separation of the leaves is conceivable. For the compressive strength of such masonry, the authors indicated that the adhesive shear strength (cohesion) between the brick and the bed joint becomes decisive.

When there is a difference in stiffness between different leaves, the load is preferentially carried by the stiffer one. As observed in Section 3, the stiffness of the mortar leaf was significantly lower than that of the thin-tile leaf. Table 4 shows the detachment compressive strength, considering that the load is carried only by the thin-tile leaves (*f_ktdc_*), i.e., the detachment load distributed by the cross-section of specimens minus the thickness of the mortar leaves. 

No substantial differences were found between the values of the two- and three-leaf masonry. This shows that since there are significant differences in stiffness, the load is mainly carried out by the ceramic leaves, and there is a very low load or no load carried out by the mortar leaves. Other authors obtained similar results [42].

From the results of this study, failure mode could be associated with shear stress in the thin-tile-mortar vertical interface, comparable to brick masonry loaded parallel to bed joints. On the other hand, due to the lateral movement of the thin-tile leaf, or its slenderness, this failure could be associated with tensile stress at the interface. Future studies will be needed to determine what stress is the precursor of the collapse.

## 5. Conclusions

The following conclusions can be drawn from the results obtained in this study:

The failure mode of thin-tile masonry is characterized by the cracking of the vertical thin-tile-mortar interface and the subsequent detachment of the leaves. From this point on, the leaves work individually. 

The stress–strain experimental behavior of thin-tile masonry, up to detachment, is as would be expected for brick masonry loaded parallel to bed joints.

The collapse of the masonry is generally reached through the buckling of the leaves. The origin of the observed phenomenon is the lateral movement of the thin-tile leaf. This movement could be associated with a loss of verticality due to its slenderness or an out-of-plane rotation of the leaf. Future studies will be needed.

The exponential formulation suggested by the standards for a brick masonry loaded parallel to the bed joints appears only suitable for a three-leaf thin-tile masonry, but with a more relevant impact of the mortar on the global compressive strength of the thin-tile masonry.

The detachment compressive strength of thin-tile masonry is the great interest. Depending on the geometry and strength of the individual leaves, higher levels of stress can be obtained, but each leaf bearing the load individually.

The load was mainly carried by the thin-tile leaves. Therefore, the role of the mortar leaf on the compressive strength seems related to the ability to hold together the thin-tile leaves. 

The failure mode of the thin-tile masonry, and due to the thinness of the individual leaves, it is not clear whether it is due to shear or tensile stresses perpendicular to the vertical thin-tile-mortar interface. This study is a part of a larger study of thin-tile masonry. In [36], the authors concluded that the shape of specimens was adequate for the compressive strength test. The specimens were built without any vertical joints in the thin-tile leaf. In the referenced work, the authors indicate that the specimens without a vertical joint have a higher compressive strength value than those with a vertical joint. It is not clear from the failure mode of the thin-tile masonry whether this joint would modify its detachment compressive strength. From the failure mode observed in this study, it is not clear whether the absence of such a joint modifies the compressive strength of the multi-leaf thin-tile masonry. Further studies of the failure mode of this masonry will be necessary, including analytical FEM models.

## Figures and Tables

**Table 1:** The mechanical properties of the thin-tile and mortar specimens.

Material	Thickness (mm)	Specimen		Compressive Strength (N/mm^2^)		Normalized Compressive Strength (N/mm^2^)	Young Modulus (N/mm^2^)	
		Identification	Samples	*f′_tt_*	*f_m_*	*f_tt_*	*E_tt_*	*E _50–30_ ^(1)^*
Thin-tile C1	18	C1 18	5	13.55 (0.22)	-	21.87 (0.22)	13,873 (0.38)	-
-	32	C1 32	5	22.35 (0.32)	-	35.45 (0.32)	10,457 (0.17)	-
Thin-tile C2	18	C2 18	5	23.07 (0.15)	-	37.24 (0.15)	5244 (0.08)	-
-	32	C2 32	5	19.67 (0.21)	-	31.44 (0.21)	4843 (0.25)	-
Cement Portland mortar		MP1	6	-	5.95 (0.16)	-	-	-
-	-	MP2	-	-	10.97 (0.15)	-	-	-
-	-	MP3	-	-	21.27 (0.08)	-	-	-
-	-	MP4	-	-	9.96 (0.09)	-	-	-
-	-	MP5	-	-	7.76 (0.03)	-	-	7976 (0.33)
-	-	MP6	-	-	4.88 (0.11)	-	-	-
-	-	MP7	-	-	9.41 (0.05)	-	-	-
-	-	MP8	-	-	5.66 (0.06)	-	-	-
-	-	MP9	-	-	3.53 (0.04)	-	-	-
-	-	MP10	-	-	3.25 (0.15)	-	-	-
Natural hydraulic lime mortar		MC1	6	-	7.11 (0.11)	-	-	-
		MC2	6	-	8.60 (0.08)	-	-	-
-	-	MC3	6	-	7.54 (0.18)	-	-	-
-	-	MC4	6	-	6.07 (0.03)	-	-	-
-	-	MC5	6	-	7.20 (0.06)	-	-	5102 (0.21)
-	-	MC6	6	-	7.15 (0.09)	-	-	-
-	-	MC7	6	-	5.95 (0.06)	-	-	-
-	-	MC8	6	-	7.07 (0.08)	-	-	-
-	-	MC9	6	-	7.08 (0.03)	-	-	-
-	-	MC10	6	-	5.40 (0.06)	-	-	-

*^(1)^* Values from [36].

**Table 2:** Mechanical properties of the prisms.

Test	Leaves	Materials		Prisms	Samples	Compressive Strength		Young	Failure
		Thin-Tile	Mortar			Peak *f_kt_* (N/mm^2^)	Detachment *f_ktd_* (N/mm^2^)	Modulus *E_kt_* (N/mm^2^)	Mode
Compressive strength	2	C132	MP	C1MP2	6	12.43 (0.34)	7.74 (0.40)	16,270 (0.21)	Buckling or crushing
	-	-	MC	C1MC2	6	8.62 (0.10)	4.99 (0.24)	18,269 (0.47)	Buckling
	-	C228	MP	C2MP2	6	10.36 (0.15)	7.18 (0.11)	11,839 (0.34)	Buckling
	-	-	MC	C2MC2	6	8.37 (0.06)	6.70 (0.09)	14,122 (0.18)	Buckling
	3	C118	MP	C1MP3	6	7.64 (0.43)	6.54 (0.43)	14,305 (0.44)	Buckling
	-	-	MC	C1MC3	6	5.30 (0.15)	4.17 (0.28)	9247 (0.54)	Buckling
	-	C218	MP	C2MP3	6	7.86 (0.27)	6.76 (0.30)	11,536 (0.39)	Buckling
	-	-	MC	C2MC3	6	7.07 (0.05)	5.94 (0.14)	10,823 (0.20)	Buckling

**Table 3:** Experimental values of coefficients *K*, *α* and *β* and the coefficient of determination (R^2^).

Thin-Tile Masonry	*K*	*α*	*β*	R^2^
Two-leaf	1498	−1.68	0.03	0.21
Three-leaf	0.16	0.57	0.84	0.72

**Table 4:** The detachment compressive strength, with the load borne for the thin-tile leaves.

Two-Leaf		Three-Leaf	
Specimens	*f_ktd_* (N/mm^2^)	Specimens	*f_ktd_* (N/mm^2^)
C1MP2	10.00 (0.44)	C1MP3	10.04 (0.40)
C1MC2	5.89 (0.23)	C1MC3	6.07 (0.25)
C2MP2	9.44 (0.14)	C2MP3	9.98 (0.31)
C2MC2	8.77 (0.09)	C2MC3	8.76 (0.17)

## Data Availability

Data sharing is not applicable to this article.
